# Hyperspectral Imagery for Assessing Laser-Induced Thermal State Change in Liver

**DOI:** 10.3390/s21020643

**Published:** 2021-01-18

**Authors:** Martina De Landro, Ignacio Espíritu García-Molina, Manuel Barberio, Eric Felli, Vincent Agnus, Margherita Pizzicannella, Michele Diana, Emanuele Zappa, Paola Saccomandi

**Affiliations:** 1 Department of Mechanical Engineering, Politecnico di Milano, 20156 Milan, Italy; martina.delandro@polimi.it (M.D.L.); ignacio.espiritu@mail.polimi.it (I.E.G.-M.); emanuele.zappa@polimi.it (E.Z.); 2 IHU-Strasbourg, 67000 Strasbourg, France; manuel.barberio@ihu-strasbourg.eu (M.B.); eric.felli@ihu-strasbourg.eu (E.F.); vagnus@orthogrid.com (V.A.); margherita.pizzicannella@ihu-strasbourg.eu (M.P.); michele.diana@ihu-strasbourg.eu (M.D.); 3 Department of General Surgery, Ospedale Card. G. Panico, 73039 Tricase, Italy; 4 Research Institute against Cancer of the Digestive System IRCAD, 67091 Strasbourg, France; 5 ICube Laboratory, Photonics Instrumentation for Health, 67400 Strasbourg, France

**Keywords:** hyperspectral imaging, image processing, image segmentation, laser ablation, tissue chromophores, thermal damage, temperature-dependent tissue chromophores, methemoglobin

## Abstract

This work presents the potential of hyperspectral imaging (HSI) to monitor the thermal outcome of laser ablation therapy used for minimally invasive tumor removal. Our main goal is the establishment of indicators of the thermal damage of living tissues, which can be used to assess the effect of the procedure. These indicators rely on the spectral variation of temperature-dependent tissue chromophores, i.e., oxyhemoglobin, deoxyhemoglobin, methemoglobin, and water. Laser treatment was performed at specific temperature thresholds (from 60 to 110 °C) on in-vivo animal liver and was assessed with a hyperspectral camera (500–995 nm) during and after the treatment. The indicators were extracted from the hyperspectral images after the following processing steps: the breathing motion compensation and the spectral and spatial filtering, the selection of spectral bands corresponding to specific tissue chromophores, and the analysis of the areas under the curves for each spectral band. Results show that properly combining spectral information related to deoxyhemoglobin, methemoglobin, lipids, and water allows for the segmenting of different zones of the laser-induced thermal damage. This preliminary investigation provides indicators for describing the thermal state of the liver, which can be employed in the future as clinical endpoints of the procedure outcome.

## 1. Introduction

Minimally invasive treatments based on percutaneous thermal ablation of the liver are considered fundamental techniques for the treatment of some pathologies, like hepatocellular carcinoma and liver metastases, when the patient cannot undergo surgery or transplant. Thermal-based treatments have the advantage of being a relatively low-risk minimally invasive procedure to be used in the treatment of focal liver tumors. Moreover, image-guided tumor ablation is recommended as the most appropriate therapeutic choice in terms of tumor local control, safety, and improvement in survival [1]. Photothermal therapy [2,3] is a minimally invasive treatment aiming at destroying cancerous tissue, through the exposure of the tissue to high temperature. Photons from low-intensity laser energy interact with the molecular chromophores present in the medium, producing a local temperature increase. Prolonged heating of tumor cells to 45–55 °C leads to cell death, whereas short exposure of cells to temperatures that exceed 60 °C causes irreversible cell damage [4,5]. The success of tumor therapy depends on the efficiency of tumor resorption while preserving the normal tissue around the target. Heating of biological tissues can produce reversible injury potentially repairable by host mechanism, and irreversible damage. Thus, desired tumor remotion with thermal treatments requires accurate control for the estimation of the produced thermal damage. In some cases, thermal treatment monitoring is based on temperature measurement. Magnetic resonance (MR) thermometry is the main image-based technique recognized in clinical practice [6]. Despite the non-invasiveness and its potential in providing two or three-dimensional information of the tissue temperature, it is costly and holds important limitations related to the motion artifacts and the necessity of working with MR-compatible devices [7]. Further temperature monitoring approaches use biocompatible invasive devices, such as thermocouples and fiber optic sensors [8,9,10,11]. Whereas the thermocouples provide only a single point measurement, the optic technology allows the measurement of temperature in different points of the tissue with a single small-sized element. However, both devices require direct contact with the target of interest, and thus the insertion in the tissue undergoing the treatment. Along with the temperature locally reached during the procedure [8,12,13,14], the exposure time is also a meaningful parameter for the thermal damage determination [15,16]. It is well known that the mechanisms at the basis of the hyperthermia-mediated cells death lead to the definition of three zones characterizing the thermal damage: a central zone, experiencing irreversible damage, a peripheral zone, characterized by reversible damage, and a third zone exhibiting no significant effects [4,17]. A proper combination of time and temperature can lead to the generation of these three zones. The tissue destruction in the central zone of the target is a sign of irreversible damage, characterized by the coagulation of cellular proteins and impairment of the tissue structural components and the vasculature. The peripheral zone experiences the sublethal thermal damage and defines the boundary of the damage, which is characterized by cells vulnerable to further injury. Beyond the effective border of tissue destruction, non-lethal temperatures mediate physiological changes such as augmentation of tissue oxygenation and blood perfusion. For the mentioned reasons, the duration of the thermal exposure should be tuned according to the size and the location of the target, as well as to the treatment strategy. In this regard, an approach for the indication and quantification of the intraprocedural thermal damage would be beneficial for therapy optimization. Indeed, the goal of thermal ablation is to achieve a treatment margin that extends just beyond the boundary of the tumor, to limit any residual malignant cells which can cause local tumor progression. Traditional imaging techniques, including contrast medium-enhanced computed tomography or MR imaging, are used after thermal ablation to detect a viable tumor. However, clear characteristics of residual or recurrent tumor remain are urgent to be clearly defined [18].

Temperature induces alteration into the tissue constituents and their structural organization, thus resulting in a measurable change of tissue optical properties. Shifts in absorption peaks, change in the index of refraction, biochemical change in hemoglobin, and variation in water content are among the effects occurring with a temperature increase, and the consequent thermal damage. Tissue chromophores such as oxyhemoglobin (HbO_2_), deoxyhemoglobin (Hb), and water (H_2_O) [19] are sensitive to the temperature of the environment, thus monitoring their spectral variation has the potential to be the basis for design a therapeutic supportive-tool, which can indicate the thermal tissue state [20,21]. In this scenario, hyperspectral imaging (HSI) holds the potentiality of combining spectral and spatial information of the thermal state of the tissue [22,23,24,25]. The use of HSI is rapidly increasing in the biomedical field, where HSI is devoted to “see the invisible” in several diagnostic and therapeutic applications [26,27]. Some of the numerous examples of the HSI potential for diagnostical purposes are the detection of cancers [28,29] by the differentiation of diverse protein biomarkers and genomic alterations [30], the digital and computational pathology [31], the non-invasive detection of skin chromophores and features [32]. Besides, HSI is also valid support for therapy and surgery guidance. It allows us to identify the anatomy and molecular content of tissue during a laparoscopic surgery of a swine [33], to visualize the chemical composition of tissue during renal surgical procedures in vivo [34], and to detect ischemia and anatomy of organs in animal models [35,36,37,38].

In this work, hyperspectral data collected during a laser ablation treatment of an in vivo swine liver were processed to identify indicators that allow for assessing the thermal therapy outcome starting from the spectral variation of different tissue chromophores. Normal in-vivo liver has been chosen as a representative model of the main physiological conditions of a regular thermal ablation procedure such as blood perfusion, body temperature, and physical properties. The approach here proposed for processing the hyperspectral images is based on three main steps: (i) a pre-processing phase, in which the absorbance images undergo the breathing motion compensation and the spectral and spatial filtering; (ii) the selection of spectral bands correspondent to specific tissue chromophores and the analysis of the areas under these spectral curves for each band of interest; and (iii) extraction of information useful to propose indicators and their application in the segmentation of images to detect the produced damage.

## 2. Materials and Methods

### 2.1. Hyperspectral Camera System

The hyperspectral camera system (TIVITA^®^, Diaspective Vision GmbH, Am Salzhaff, Germany) used in this study was equipped with a push-broom imaging spectrometer with a slit-shaped aperture, internal stepper motor moving the slit of the spectrograph, high quality infrared enhanced CMOS, and data processing equipment [39]. The acquisition of a single hyperspectral data cube, i.e., the hypercube, was performed with a camera-specific module of the Perception Studio software (Perception Park GmbH, Graz, Austria). An RGB image was also provided in each acquisition time. The spectral range of this camera is 500–995 nm, and the final hypercube resolution was 640 pixels × 480 pixels × 100 wavelengths. Acquisition distance was typically between 20 and 60 cm from the measured area, adjustable to the specific measurement conditions. The light source was a 20 W OSRAM Halospot 70 halogen lamp (OSRAM GmbH, Munich, Germany) allowing for intense, broadband, temperature-stable, homogeneous, and fast pulses radiation. The calibration of the wavelength was performed during camera production. Furthermore, dark current effects are corrected after the recording of the data cube by the developed software component. The camera collected and processed the information from the electromagnetic spectrum measuring the reflectance spectra generated by the object of study. To convert image data from radiance to relative reflectance, a white reference object characterized by a high diffuse reflectance was used to create a reference cube before the measurements started; this also balanced regional differences in lighting [40].

### 2.2. Animal Model Experimental Protocol

**Figure 1:**
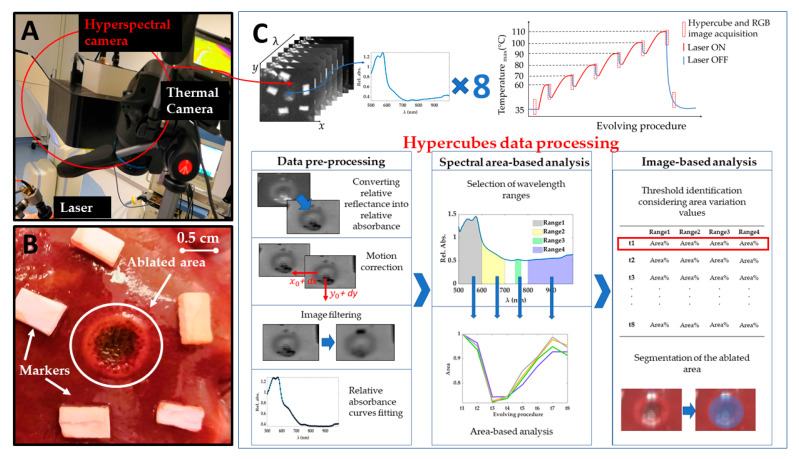
(**A**) Experimental setup including laser system delivering light at 808 nm, thermal camera acquiring temperature values during the overall procedure, and hyperspectral camera. (**B**) A detail of one target area (Test 1) after the treatment. Two different regions are visible for the ablated area: (i) a darker center and (ii) a brighter ring zone highlighting boundaries of the area of interest. (**C**) A scheme showing the acquired hypercubes, the workflow of the experiments, and the data processing steps for the hyperspectral data.

Experiments were carried out at the Institute of Image-Guided Surgery of Strasbourg, France. The study received full approval from the Institutional Ethical Committee of the Institute of Image-Guided Surgery of Strasbourg (Protocol N°38.2015.01.069), and the French Ministry of Superior Education and Research (Protocol N° APAFiS##19543-2019030112087889). It was conducted in compliance with French laws for animal use and care and according to the European directives (2010/63/EU). One female pig (Large White, weight 34.6 kg) received intramuscular ketamine (20 mg/kg) and azaperone (2 mg/kg) (Stresnil; Janssen-Cilag, Beerse, Belgium) for premedication. Induction was achieved with intravenous propofol (3 mg/kg) combined with rocuronium (0.8 mg/kg). Anesthesia was maintained with 2% isoflurane. Afterward, the pig was sacrificed with an intravenous injection of pentobarbital sodium (40 mg/kg) (Exagon^®^, AXIENCE, Pantin, France), under a 5% isoflurane anesthesia. After the laparotomy, exposure of the liver was achieved and two distinct areas on the liver surface underwent the treatment. Near-infrared laser light at 808 nm was used to perform a contact-less ablation of the liver surface. The laser spot was around 1 cm in diameter producing ablated areas of around 1 cm. The procedure was recorded with a thermographic camera (FLIR T540 (FLIR Systems, Wilsonville, OR, USA), 464 pixels × 368 pixels spatial resolution, 2 °C accuracy) capturing images of the scene at 10 fps. The real-time measurement of the temperatures in the area of interest, the ablated zone on the liver surface, allowed the control for chosen temperature thresholds used as indicators of produced thermal effect. These thresholds were selected as maximum temperatures measured within the target zone. The hypercubes (640 pixels × 480 pixels × 100 wavelengths each) were collected before starting the ablation (liver initial temperature), for six temperature thresholds relevant to study the thermal tissue state (i.e., 60, 70, 80, 90, 100, 110 °C), and 5 min after the procedure, for a total of 8 hypercubes for each test. An RGB image was also acquired for these 8 acquisition times. Besides, the laser current (3000 mA) was also controlled under set temperature thresholds. Once the set temperature threshold was reached, the laser system was switched off, and the acquisition of the hyperspectral images was performed in around 6 s. Figure 1A reports the setup consisting of a laser applicator, and thermal and hyperspectral cameras. Figure 1B shows a detail of the target area after the treatment, where peripheral and central damage zones are distinguishable along with markers surrounding the lesion. Markers of polyurethane material were used as references for correcting the breathing motion errors affecting images. The treatment was performed on two different zones of the liver surface for a total of two laser ablation tests producing two ablated areas. The total duration of the two procedures was different due to the different features of the two treated zones: ~12 and 16 min for Test 1 and Test 2, respectively. The two target areas were chosen under manufacturer guidelines of the HSI camera use. The TIVITA^®^ camera was perpendicularly adjusted with a distance of 40 cm, ensuring as little as possible external light irradiations on the measuring area [39]. For this purpose, all the light sources in the surgery room, except for the camera’s lamps, were switched off during the hyperspectral acquisition.

The experimental strategy adopted in the present work and clarified in the next section is described in Figure 1C.

### 2.3. Hypercubes Data Processing

The processing of the data was performed off-line on Matlab R2020a^®^ (MathWorks, Natick, MA, USA) and developed into three main stages, as shown in Figure 1C: pre-processing, spectral area-based analysis, and, finally, image-based analysis.

#### 2.3.1. Data Pre-Processing

The pre-processing phase includes four steps: the transformation of spectral data into absorbance data, the compensation of the motion of the lesion due to mechanical ventilation, and image filtering and fitting. In the first step, relative reflectance data provided by the camera are converted allowing to perform the analysis on the resulting relative absorbance [40].

During the ablation procedure, the pig was anesthetized and mechanically ventilated. Even though the animal’s breath was artificially stopped for a few seconds during the HSI acquisition, the previous breathing motion caused a displacement of the ablated region within the collected hyperspectral images. To compensate for movement, an algorithm able to compensate for the effect of the breathing motion of the animal on images is applied. The aim is to obtain images where the pixel under analysis always corresponds to the same point of the pig organ for all the hypercubes, and, consequently, reduce the variability in the spectral curves acquisition process. The HSI camera is fixed, looking on the top, normal to the liver surface and the liver is not deformed thanks to open surgery hence we could assume in the HSI view a 2D rigid motion. To compensate for the motion it is first necessary to estimate the vertical and horizontal coordinates following the displacement of the ablated region. This is done using a Pattern Matching algorithm. To improve the accuracy of the estimated displacements, the pattern includes not only the ablated region but also a portion of the bright markers around it, visible in Figure 1B. The high contrast between the markers and the background tissue is fundamental for the accurate estimation of the displacements, in particular in the first part of the treatment, where the lesion is still hardly visible. Moreover, the markers are visible in all the planes of the hypercubes, since their absorbance is significantly different from the absorbance of the surrounding tissue for all the wavelengths in the measuring range of the adopted camera. Thanks to this property, it is possible to estimate the motion by applying the pattern matching to any plane of all the hypercubes. As soon as the position of the pattern is estimated for each hypercube (i.e., for each step of the treatment), the cropping is straightforward, since it is sufficient to set the cropping rectangle according to the displacement computed previously. In this way, the center of the lesion always appears at the center of the cropped image. The cropping rectangle should be small enough to avoid including regions close to the border of the liver, to ensure that all the points within it follow the same motion. Moreover, it should be large enough to include the lesion and surroundings areas. The final size of the cropping rectangle was chosen as 125 pixels × 125 pixels corresponding to ~35 mm × 35 mm. The motion algorithm function returns a set of cropped datacubes, thus, the motion is compensated and the data to be processed thereafter are greatly reduced, along with the computational time required for the subsequent analyses.

Due to unavoidable experimental disturbances, the data in the hypercubes are affected by random noise. To mitigate the noise, we extracted from a hypercube the planes associated with each wavelength and filtered them with image processing tools. For the filtering, each image plane has been convolved with a spatial filtering mask (filter kernel) [41]. The selected filter is a bidimensional circular correlation kernel, which matrix size is 2∙radius + 1. The parameters of the filter, such as the radius of the disk, i.e., the size of the bidimensional filter, must be tuned to obtain a filter size large enough to effectively reject the noise. On the other hand, it is fundamental to avoid that the information stemmed from pixels that are not from the area of interest can alter the data of the pixel to study. Moreover, increasing the size of the filter can attenuate the contrast between the region under laser treatment and surrounding areas. Thusly, the tuning of the filter parameters, described in Section 3.1, is a trade-off between noise reduction and spatial selectivity of the filtered image.

While image filter reduces noise working on neighbor pixels belonging to the same wavelength plane, curve fitting is used here to fit the absorbance curve of every single pixel along the wavelength axes. Absorbance curve fitting allows to reduce noise and to create a continuous model of these curves to perform further quantitative analyses. Cubic spline has been adopted for the fitting since it allows following the trend of the absorbance curves in all the spectral ranges of the wavelength of interest for this work. Moreover, the computation time for cubic spline fitting is negligible concerning the other computation steps and does not prevent the real-time implementation of the technique.

#### 2.3.2. Spectral Area-Based Analysis

In this second analysis, pre-processed data were used to study the optical behavior for specific spectral ranges during the treatment. The areas under the spectral curves were calculated for this purpose to select possible tissue thermal state indicators.

**Figure 2:**
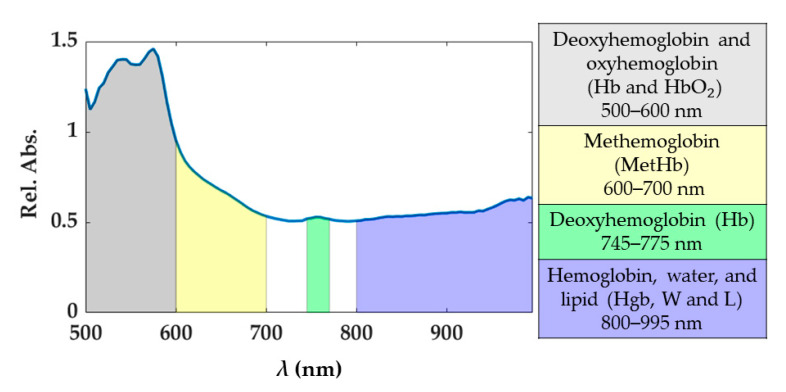
Spectral ranges considered for the study. These spectral ranges are associated with tissue chromophores.

Under normal conditions, oxyhemoglobin (HbO_2_) and deoxyhemoglobin (Hb) are the principal chromophores of blood showing sharp absorption peaks [19]. The main blood spectral absorbance range falls within the 500 and 600 nm, though there is a visible Hb peak at approximately 760 nm. In this range, HbO_2_ presents a pair of peaks at 525 and 575 nm, while Hb at 540 nm approximately. The presence of blood in the part of the tissue analyzed would be distinguishable when two peaks appear in the spectral curve, as the sum of the absorption curves of both Hb and HbO_2._ The third type of hemoglobin, methemoglobin (MetHb), is produced by photo-induced oxidation of hemoglobin. The formation of MetHb is thermodynamically favorable and irreversible, but there is a barrier to the reaction requiring some minimum activation energy (heat) for initiation [42]. MetHb is formed at temperatures above 65 °C, and its presence in the spectral curves can be most noticeable in the spectral range of 600 to 700 nm (peak at approximately 630 nm). Also, in the range 800–995 nm the hemoglobin contribution cannot be excluded, and here broad peaks for the MetHb and HbO_2_ are present [19]. Conversely, the water absorption spectrum is higher over the near-infrared region, being nearly transparent in the visible range. Therefore, the presence of water in the tissue analyzed would appear in the spectral range from approximately 700 to 995 nm. Also, for the fatty tissues, the lipids show a dominant absorption peak at 930 nm [43]. Previous studies have shown absorption spectra variation in shape and magnitude for water, Hb, and HbO_2_ when a temperature increase occurs [20,21,44]. For instance, a decrease in Hb absorption with increasing temperature is expected, as well as a bathochromic shift of HbO_2_. Furthermore, in the region 540–580 nm, the absorptivity may increase or decrease depending on the magnitude of the temperature increase. To account for the potential and physiological-related uncertainty in the measured spectra, we chose to use four spectral ranges described in Figure 2. Each range was associated with the chromophores showing the predominant response within the range itself. The 800–995 nm is a complex range including hemoglobin, water, and lipid contributions. Indeed, the single spectral responses of these chromophores are not easily distinguishable in this spectral range. Also, the generic hemoglobin (Hgb) term is involving the contributions of Hb, HbO_2_, and MetHb in the optical response when a temperature increase is occurring.

**Figure 3:**
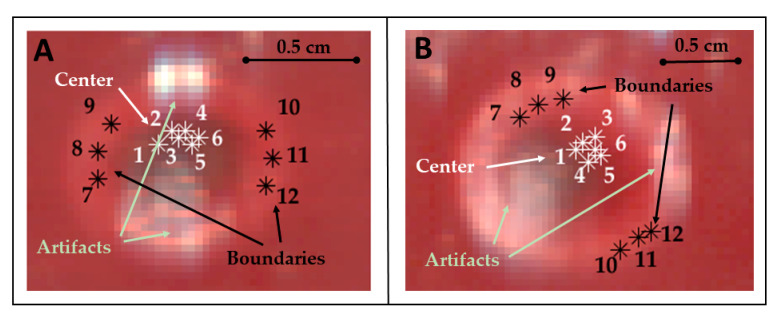
Pixels of the center and boundary of the lesion selected for studying the area under the spectral curves (spectral integrals) in Test 1 (**A**) and Test 2 (**B**). Pixels were selected in zones not affected by artifacts. Positions chosen for these pixels are highlighted using RGB images collected by the HS camera at the end of the treatment and showing the thermal damage.

The thermally-damaged zone has been explored by analyzing the spectral information in correspondence with the center and boundary of the target. In particular, the study involved the calculation of the areas under the spectral curves for the four wavelength ranges (hereinafter referred as spectral integrals). The analysis has been performed for points belonging to both the center and boundary of the target and for each test (Figure 3).

For obtaining the values of the spectral integrals for the optical response in the center of the target, the following steps have been performed: (i) selection of 6 pixels belonging to the center of the lesion, in the specific the center was distinguishable in the images and the pixels were manually chosen within the central zone; (ii) extraction of the spectral curves from these pixels and, in these spectra, (iii) selection of the wavelength range of interest for each chromophore; (iv) calculation of the areas under the curve’s range, using the integration of the curve model. We refer to this area calculated as integral as $A_{t_{i}}$, where $t_{i}$ represents the specific acquisition time; (v) normalization of $A_{t_{i}}$ values, dividing them by the corresponding initial values (i.e., $A_{t_{0}}$) extracted from the hypercube acquired at 35 °C; (vi) estimation of the mean and the relative uncertainty among the 6 pixels, obtaining at the end a normalized area ($NA_{t_{i}}=\;\frac{A_{t_{i}}}{A_{t_{0}}}$) for each spectral range of interest. This process was repeated for each acquisition time ($t_{i}$), giving at the end the temporal evolution of the normalized area of the four chromophores, during the overall procedure. The same procedure was repeated for a set of pixels at the boundary of the region, to allow for a comparison between the results in the center and at the boundary of the target.

Furthermore, to identify possible indicators for thermal state detection, the normalized area $NA_{t_{i}}$ was used to obtain a normalized area variation measured for each chromophore as follows:

(1) $$\Delta NA_{t_{i}}=NA_{t_{i}}-NA_{t_{0}}=\frac{A_{t_{i}}}{A_{t_{0}}}-1$$

where the normalized area for starting acquisition time $NA_{t_{0}}$ is equal to unitary value.

The repeatability and the accuracy of the implemented approach were assessed by analyzing the hyperspectral information obtained in two different and not-overlapped laser-ablated regions of the hepatic tissue. The two tests were first analyzed separately and then merging all the data. In the latter case, the standard deviation for each $\Delta NA_{t_{i}}$ was computed among the 12 pixels (6 pixels of Test 1 + 6 pixels of Test 2) belonging to each zone of interest. Results are reported in Section 3.2.

#### 2.3.3. Image-Based Analysis

At first, a normalized image variation ($\Delta NI_{t_{i}}$) was calculated following these steps: (i) selection of the images previously cropped (after motion correction process) in the four spectral range of interest; (ii) averaging of the images within the range for obtaining a single image for each chromophore ($I_{t_{i}}$); (iii) normalization of the images dividing them by initial ones corresponding to acquisition at 35 °C ($I_{t_{0}}$); (iv) subtraction of the normalized image $NI_{t_{i}}$ to the $NI_{t_{0}}$ thus obtaining an image showing the specific chromophore variation concerning its initial condition during the overall procedure.

(2) $$\Delta NI_{t_{i}}=NI_{t_{i}}-NI_{t_{0}}=\frac{I_{t_{i}}}{I_{t_{0}}}-NI_{t_{0}}$$

A preliminary evaluation of the potentiality of using measured indicators for defining the outcome of the thermal treatment was performed through an image segmentation process. In the specific, the abilities to detect damaged area visible in the RGB images collected at the end of the procedure was tested segmenting HS images using a thresholding technique. Thresholds were chosen from the obtained ΔNA values corresponding to the variation found for specific spectral ranges during the treatments. Since an irreversible and instantaneous thermal damage is expected to occur at 60 °C, the ΔNA calculated at such maximum temperature and for the four spectral ranges were identified as thresholds for catching the damage in the RGB images.

The segmentation was then applied to the image collected at 110 °C showing the final thermal outcome and for each tissue chromophore. The final results were computed by combining the information gathered for different tissue chromophores.

## 3. Results

### 3.1. Results of the Data Pre-Processing

**Figure 4:**
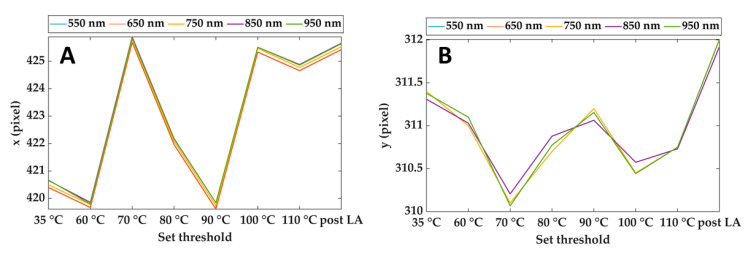
Horizontal (**A**) and vertical (**B**) coordinates of the reference point (center of the cropping rectangle) for different wavelengths and during the treatment. Results refer to Test 1.

Figure 4 shows an example of the output of the Pattern Matching: *x* and *y* coordinates of the center of the cropping rectangle for different spectral images and each step of the treatment. As is visible, the maximum discrepancies between the pattern positions estimated at different wavelengths are of the order of 0.1 pixels Furthermore, the maximum displacement of the *x* coordinate during the overall treatment is around 6 pixels, whereas it reaches 2 pixels for the *y* coordinate. Considering that the purpose of these data is to compensate for the motion of the lesion due to mechanical breathing, the results ensure proper tracking of the lesion during the overall procedure and for the full wavelength range.

**Figure 5:**
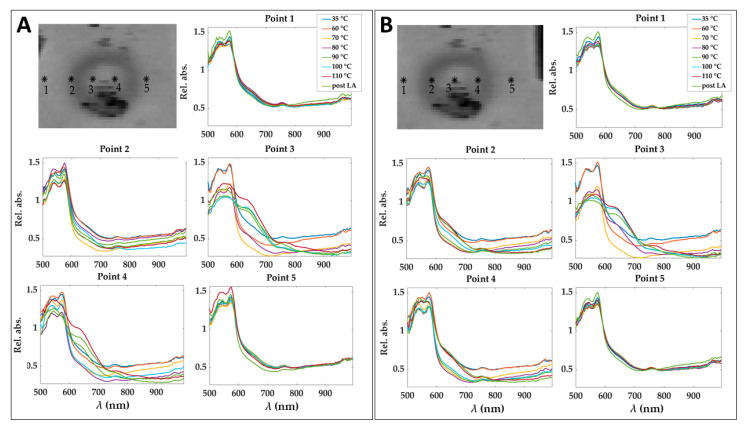
Spectra obtained for 5 pixels in the relative absorbance cubes for the eight acquisition times before (**A**) and after (**B**) applying motion compensation step. Pixels positions are shown before and after the correction of a cube acquired at 90 °C and for the image at 750 nm as an example. Results refer to Test 1.

To show the motion compensation results, a comparison among the spectra extracted in 5 pixels of relative absorbance cubes before and after the application of the motion compensation algorithm is reported in Figure 5. The pixels are placed at different positions in the damaged zone. The effect of the motion correction is evident in correspondence of Point 4, where spectra behavior consistently changes because of point shifting from the central damaged zone to the periphery.

**Figure 6:**
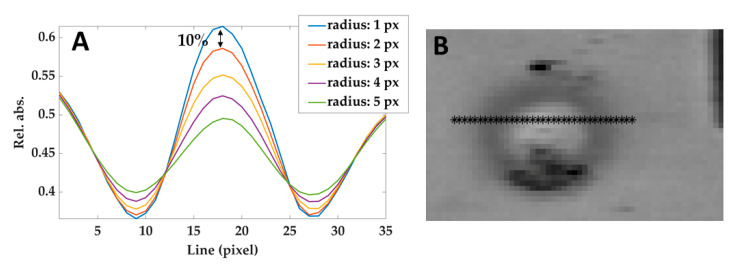
(**A**) Relative absorbances profiles taken from a line of pixels crossing the lesion (shown in (**B**)) in the image filtered using different radius. The black arrow shows the difference between the curves obtained by using 1 pixel-filter and 2 pixels-filter and defining the maximum acceptable reduction of 10%. Similar results have been obtained at different temperatures and hypercubes.

The effect of the correlation kernel disk filter used in this phase can be tuned by modifying the radius: the larger is the radius, the stronger is the noise rejection. However, large radius values create a blur-like effect and therefore affect the contrast (i.e., the difference between the brightness of different areas). Hence, the procedure to choose the value of the radius was: (i) measuring the reduction of the contrast between the center of the lesion and the non-treated areas in the surroundings, as a function of the filter radius (see Figure 6A); (ii) heuristically selecting the maximum values that do not reduce the contrast of more than 10%. According to this criterion, a radius of 2 pixels has been adopted.

### 3.2. Results of the Spectral Area-Based Analysis

**Figure 7:**
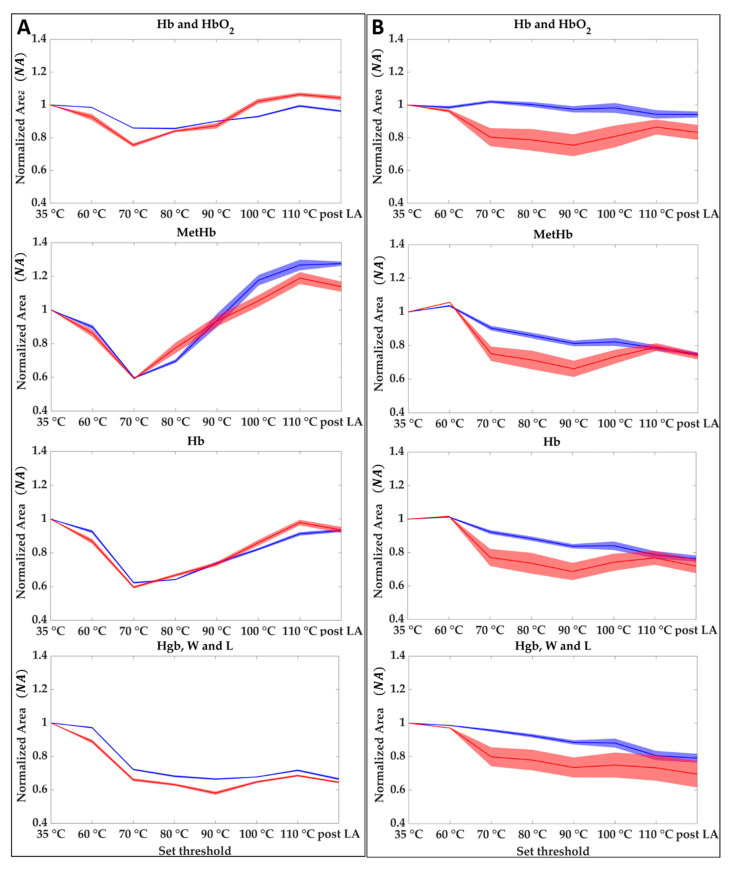
Hb and HbO_2_, Hb, MetHb, and Hgb, W and L at different set thresholds for central (**A**) and boundaries pixels (**B**). Each graph shows the averaged area trends (normalized mean area values and their uncertainty) for Test 1 (blue) and Test 2 (red).

For each of the two tests, normalized area results (NA) obtained as described in Section 2.3.2 are shown in Figure 7 for the center (**A**) and the boundary (**B**) of the lesion. The evolution of the NA during the overall treatment for the four spectral regions, Hb and HbO_2_, MetHb, Hb, and Hgb, W and L is shown. Results show that when the temperature reaches values above 90 °C in the central zone, the oxyhemoglobin disappeared at the cost of the deoxyhemoglobin and methemoglobin. On the other hand, there is an appreciable reduction in the 800–995 nm probably linkable to the water evaporation. Normalized area values for the central zone of the two tests show higher reproducibility comparing the results obtained from the analysis of the boundaries. For the boundary case, although the area values follow the same trend in both tests, the drops and variations have different values. For monitoring the ablation procedure starting from the evolution of the NA of the four chromophores it may be possible to identify the temperature steps and the level of damage reached as follows:

- 35 to 60 °C—Decrease of Hb, MetHb, and Hgb, W and L for the central zone, whereas in the boundary the thermal effect is delayed because of the heat conduction towards the peripheral area. Starting from a value equal to 1 at 35 °C, MetHb and Hb, especially, reached 0.88 and 0.89 values in the center zone;
- 60 to 70 °C—Decrease of the four chromophores for the total area (center and boundary). In the center zone, minimum values of 0.80, 0.59, 0.61 are reached for Hb and HbO_2_, Hb, MetHb, respectively. In the boundary, NA values experience a more moderate decrease with a minimum for the MetHb of around 0.82;
- 70 to 80 °C—Increase of Hb and HbO_2_, Hb, MetHb in the center, whereas for Hgb, W and L a decrease is still visible. The boundary values show still a slight decrease;
- 80 to 90 °C—Increase of Hb and HbO_2_, Hb, MetHb in the center, whereas for the boundary a decrease is still visible until reaching minimum values of 0.86, 0.73, 0.76 for Hb and HbO_2_, Hb, and MetHb, respectively. On the other hand, Hgb, W and L reaches the minimum value of 0.62 in the center.
- 90 to 110 °C—Increase of Hb and HbO_2_, Hb, MetHb. Whereas for Hb and HbO_2_, and Hb the *NA* values return almost to the initial conditions, the MetHb reached a maximum value of 1.32 following the activation of MetHb at 65 °C [42]. The final value of the area after the LA lies above the initial conditions, thus showing that the chromophore formed due to the temperature effect remains after the thermal treatment. Even the Hgb, W and L range shows a slight increase reaching 0.70 value at the end of the ablation process. In the boundary, values show a slight increase in this step. For the MetHb, the final values are below the initial conditions contrary to the situation at the center.
- 110 °C to post LA—Once a maximum temperature of 110 °C is reached the amount of chromophores in both the zones does not experience any consistent variation. A decrease is noticeable as an overall trend.

Moreover, the percentage of normalized area variation ($\Delta NA_{t_{i}}$ %) is listed in Table 1 for the four spectral regions (columns) and the entire procedure (rows) to identify the amount of change in the chromophore content indicating the specific level of thermal damage produced. For this purpose, the analysis was focused on spectral behavior during the temperature increase time to consider the LA treatment progress. In Table 1 the data from the two tests are merged and analyzed together.

The ablation procedures were performed in two different liver areas with different exposition to the HSI camera, and different physiological conditions (e.g., blood content and perfusion). These elements can be among the causes of the differences among the tests, especially in the boundary zone where the spatial gradients of the effects of the treatment are larger, leading to high standard deviation values. Even though the authors paid attention to avoiding specularity artifacts when selecting the pixels for the analysis, these artifacts in the images can affect the repeatability of the method. Furthermore, standard deviation values are also consistent for the MetHb in the central zone when a temperature of 90 °C is reached. This is because at this temperature MetHb increases until achieving a value close to the initial condition. Nevertheless, this is not limiting the use of MetHb chromophore as tissue damage indicator. Indeed, such a level of damage may be detected taking into account the procedure timeline. A reversion to the initial condition of MetHb content for a temperature higher than 70 °C defines the 90 °C step. Besides, to overcome the low repeatability issue, information among different chromophores can be combined towards a more reliable strategy to monitor the ablation procedure.

### 3.3. Results of the Image-Based Analysis

**Figure 8:**
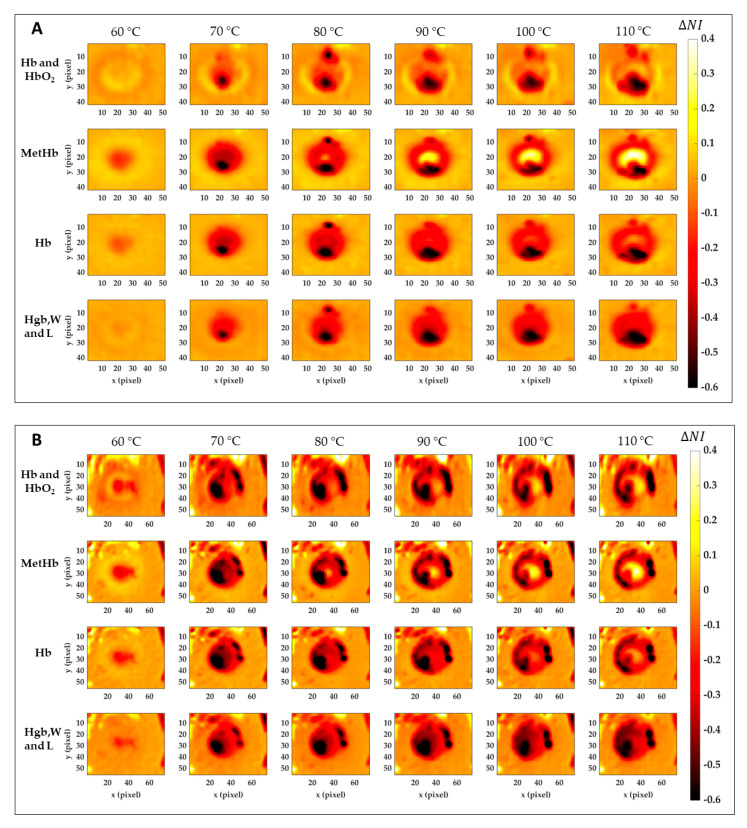
Normalized image variation for the four peculiar spectral ranges corresponding to Hb and HbO_2_, MetHb, Hb, and Hgb, W and L. Images are obtained with respect to the initial condition for Test 1 (**A**) and Test 2 (**B**) for the six temperature thresholds.

Figure 8 reports the results for the normalized image variation ($\Delta NI_{t_{i}}$) showing the specific chromophore variation due to the temperature increase and compared to its initial condition. Data here reported are 2D maps referred to the data listed in Table 1, which were found for specific pixels in both center and boundary. The different behavior for the four tissue chromophores highlights the variety of information that can be gathered from each spectral region. Also, the gradual variation for the single tissue element proves the chance to detect a specific thermal state during the treatment as well as the level of damage reached at the end of the procedure. The lower reproducibility in the boundary of the lesion, already noted in Figure 7, is probably due to low $\Delta NI_{t_{i}}$ values in Test 2. Black pixels are due to artifacts in the images. Nevertheless, comparing results for Test 1 and Test 2, a similar trend is noticeable. Hence, for both tests, the MetHb values allow for the distinction between the two levels of damage found for the center and periphery. Similar but less evident results are visible for the Hb. Hgb, W and L range, instead, shows the chance to detect the whole damaged area, helping in the border definition when preserving the surrounding healthy tissue is essential. For the Hb and HbO_2_ in the 500–600 nm range, less clear and informative content can be observed.

**Figure 9:**
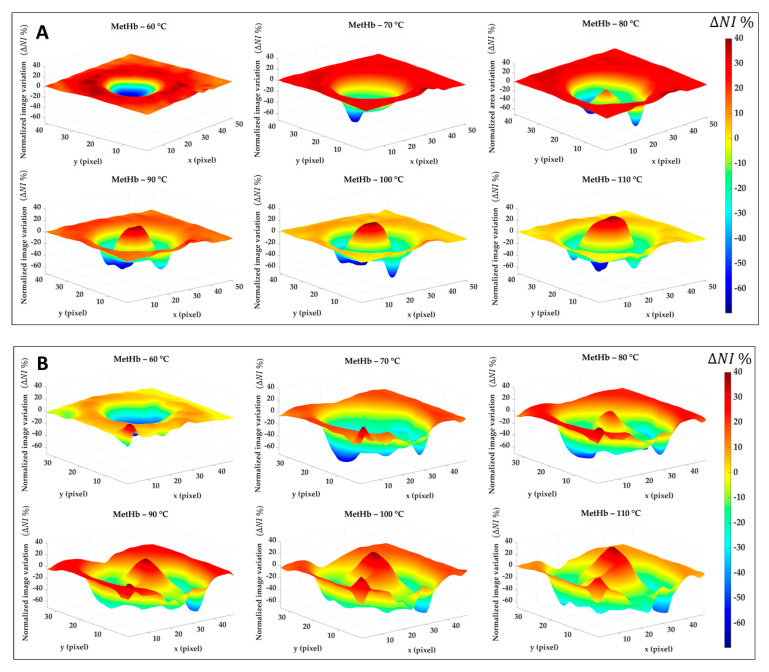
Percentage of normalized image variation ($\Delta NI_{t_{i}}$ %) in the MetHb spectral range for Test 1 (**A**) and Test 2 (**B**) during the ablation process.

Similar results are shown in Figure 9. This time, the percentage of normalized image variation ($\Delta NI_{t_{i}}$) is exhibited in 3D only for MetHb chromophore during the ablation treatment and again for Test 1 (Figure 9A) and Test 2 (Figure 9B). If in the boundary zone the MetHb is decreasing during the procedure evolution, for temperature >70 °C a central damage zone subject to MetHb formation starts to appear. The negative peaks (in blue) are due to the artifacts in the images.

**Figure 10:**
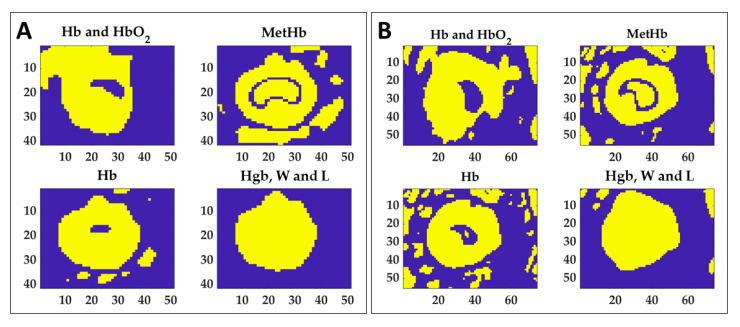
Extracted masks for the four ranges using thresholding technique in order to segment the normalized image variation acquired at 110 °C. Thresholds were chosen as normalized area variation values found at 60 °C (see Table 1) for both boundary and central zones. Results for Test 1 (**A**) and Test 2 (**B**) are reported.

To preliminarily evaluate the ability of the indicators in the thermal damage detection, normalized area variation for the 60 °C time acquisition ($\Delta NA_{t_{1}}$) calculated for the four spectral ranges of interest were set as thresholds to find the lesion. In the specific, mean values between the two tests for both boundary and central were used as indicators. The results of the segmentation process for the two tests are shown in Figure 10. Normalized image variation computed at 110 °C ($\Delta NI_{t_{7}}$) in each spectral region was segmented using the relative thresholds values to detect the total thermal outcome and specific masks were determined.

**Figure 11:**
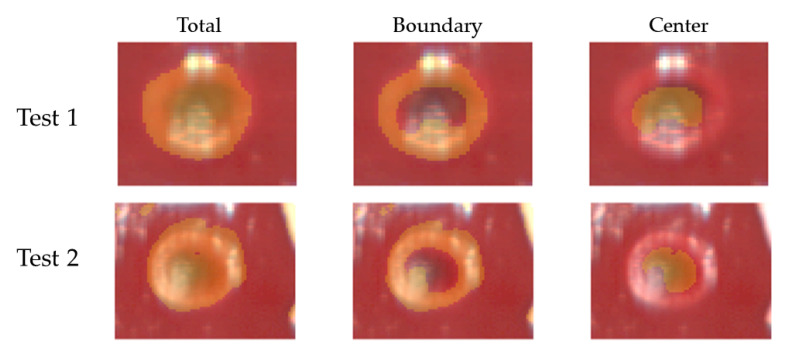
Results of segmentation for Test 1 and Test 2. Masks extracted using the thresholding technique were used to segment total damaged, boundary, and center zones in the RGB images acquired by the hyperspectral camera at the end of the ablation procedure and clearly showing the thermal outcome.

Figure 10 shows that each mask can detect different information about the produced damage. For instance, if the MetHb indicator may be used to distinguish the central zone from the periphery, the value extracted for the Hgb, W and L range can delineate the boundary of the whole target. By combining the information provided by the masks of Hb, MetHb, and Hgb, W and L, the thermally damaged zone can be detected. By summing the mask obtained for Hb and the mask of MetHb, the total burnt area can be detected for both tests, as shown in Figure 11. Besides, the background can be removed by adding the mask obtained for the last spectral range. The detection of the damaged area achieved with the proposed approach has been verified by overlapping the segmented area to the RGB image collected at the end of the procedure, thus showing the final thermal effect. Finally, the mask of the MetHb indicator has been used to identify the central zone from the boundary.

## 4. Discussion

This work presents the analysis of the thermal response of a porcine hepatic tissue induced by laser. The analysis is based on spectral and spatial information provided by HSI, in correspondence with different thermal states of the tissue. The main goal of the work is to expand the investigation of hyperspectral imaging as a future tool for monitoring the outcome of thermal ablation procedures [26]. After the stage of image filtering, motion correction, and extraction of spectral integrals corresponding to thermal-sensitive chromophores, indicators of the thermal damage were proposed. A preliminary validation step using these identified indicators was also proposed to detect thermal damage. Whereas in some previous studies the most relevant information from the original hyperspectral data is performed with a Principal Component Analysis (PCA) and its variants [45], here the features were extracted starting from theoretical knowledge of the spectral characteristic ranges where a variation due to temperature increase is expected to occur. Besides, a specific dataset allowed for easy cropping of the area of interest in the images largely decreasing the initial amount of raw data collected. Indeed, the ablated area is visible once a maximum temperature of 60 °C is reached in the tissue.

Starting from the spectral area-based analysis, results show a strong variation to be localized in MetHb, Hb, and Hgb, W and L ranges. The resultant overall trend in the central zone for MetHb and Hb consisting of an initial decrease (60–70 °C) continued by an increase for temperature >80 °C until reaching a plateau phase was also found in [24], thus confirming the main expected spectral variations for an in-vivo liver undergoing laser ablation. On the other hand, the Hgb, W and L range experienced mostly a continuous decrease, thus demonstrating a trend similar to the water peak one at 960 nm, observed in [24]. A similar analysis was performed in [46] where diffuse reflectance spectroscopy (DRS) is used for assessing the efficacy of a RadioFrequency (RF) ablation. In this case, reflectance spectra were acquired using a probe connected to the light source and a second fiber connected to a spectrometer working in the range between 400 and 1050 nm. The invasive spectral acquisition is performed since a spectroscopy needle needs to be inserted into the tissue of interest. A pronounced increase in absorption between 600 and 700 nm was observed for the colorectal liver metastases undergoing RF. Therefore, MetHb was selected as a possible marker of the thermal damage degree produced during RF ablation. In contrast with our study, direct quantification of the observed MetHb variation during the treatment was not performed. Nevertheless, the potentiality of correlating chromophore content and damage extension was already highlighted. More recently, a correlation was found between relative changes in absorption and scattering properties and the histology for an ex vivo porcine liver resulting in a classification of the thermal damage scores with an overall accuracy of 72.5% at temperatures up to 75 °C [47].

Concerning the ability to visualize and assess the produced thermal outcome, previous studies have investigated the use of HSI images. Swift et al. [48] proposed the autofluorescence HSI for myocardial scar identification in vivo. After selecting the spectral ranges experiencing a consistent variation for the pixels belonging to RF scar, they were able to identify the thermal effect through an HSI composite image computation. On the other hand, in [25] HSI was investigated to build up an optical imaging system capable of characterizing an RF thermal effect in ex-vivo liver. In this case, the authors selected 720 ± 19 nm as the optimum wavelength to discriminate among the different ablation zones, and on the chosen image they successfully captured the contours for the areas of interest using a K-mean image clustering algorithm. In the present work, hyperspectral data potentiality to furnish a complex data structure holding spectral and spatial information was exploited by computing both spectral area-based and image-based analyses. Hence, if investigating the normalized are variation ($\Delta NA_{t_{i}}$) during the procedure led to indicators extraction, computing the normalized image variation ($\Delta NI_{t_{i}}$) allowed us to preliminarily validate abilities of such indicators in the thermal outcome detection. The normalized area variation values found at 60 °C for the four spectral regions were identified as markers and used in the image analysis step. In the end, only values for MetHb, Hb, and Hgb, W and L ranges were found to be valid indicators to select damaged zones after implementing the segmentation step. Compared to previously mentioned studies, the present analysis can benefit from a combination of information for several tissue contents for a proper thermal outcome assessment. Additionally, the direct spectral response-degree of damage nexus enabled by the HSI-thermal camera combined use supports the application of the proposed strategy for thermal therapies monitoring purpose in clinical settings. Even though the spectral characteristics of normal and tumor liver can differ up to 50% [49], the approach proposed in our study should not be significantly affected by the optical properties of the reference tissue. Indeed, here we evaluate the change of chromophore-related spectral information with respect to the initial state of the tissue.

Besides its potentiality, important limitations of the HS camera use can be found in the artifacts due to specular light, mainly due to the bright and wet liver surfaces, long scanning time, and the need for proper scene illumination.

The choice of performing this preliminary investigation on the areas obtained as integrals in the four spectral ranges holds the limit of combining the effects within a large wavelength range, thus losing specific spectral information. For example, the 800–995 nm was set as a single range even though hemoglobin, water and lipid demonstrate optical contributions, especially in the case of fatty live tissues. Nevertheless, a previous work [24] showed a similar trend for the 800–995 nm thus proving not consistent loss of information in combining the overall data included in the range. This point has been partially confirmed by observing for the global Hgb, W and L range a behavior close to the single water peak measured at 960 nm [24]: here, the spectral value in the range drops with temperature, and is maintained until the end of the procedure. This result is probably attesting the dominance of the water variation in this range during thermal therapy. Indeed, previous studies showed changes in optical properties mainly for hemoglobin and water chromophores with temperature increase [19], thus confirming an insubstantial change for the lipid content. Moreover, the necessity to attempt a more robust analysis based on optical information extracted from multiple wavelengths aimed at minimizing the potential errors was associated with a single wavelength [24]. Furthermore, considering only four spectral information limits the band number for the analysis. This choice reduces the amount of data to be managed for the analysis and, at the same time, suggests the future opportunity of employing a multispectral imaging system, that has a limited number of bands, usually fewer than 10. This would lead to significant advantages since multispectral cameras are by far cheaper than hyperspectral ones, and they can manage to acquire the full image in a shot, avoiding the issue of scanning time.

Nevertheless, further studies are required concerning a more specific optical analysis and involving additional data to further confirm the repeatability and the feasibility of the results here found. In the future, the optical response for the tissue should be analyzed in detail taking into account all the spectral features to better investigate the spectrum behavior for all the tissue chromophores during the thermal treatment (especially for the 800–995 nm range) and to link the spectral variations to the physiological event occurring with temperature increase.

## 5. Conclusions

In this work, the thermal response of the hepatic tissue undergoing laser treatment is assessed through hyperspectral imaging. A combined spectral and image-based approach was developed to identify hyperspectral-inspired indicators of the liver’s thermal outcome. This approach can be potentially used for evaluating the spatial dimensions of the treated region and its margins, thus supporting the decision-making process of the clinician performing the thermal treatment. In the future, the collection of a large amount of information could provide an extensive database useful to further validate the proposed approach, and to extend the analysis also on other organs.

## Figures and Tables

**Table 1:** Normalized area variations (%) measured during the laser ablation for the center (C) and the boundary (B) of the lesion. Reported values are obtained averaging results for the two tests.

	Hb and HbO_2_	MetHb	Hb	Hgb, W and L
Set Threshold	$\Delta NA_{t_{i}}\;\%$	$\Delta NA_{t_{i}}\;\%$	$\Delta NA_{t_{i}}\;\%$	$\Delta NA_{t_{i}}\;\%$
60 °C	C = −4.47 ± 4.40 B = −2.60 ± 2.46	C = −12.02 ± 4.59 B = 4.67 ± 1.45	C = −10.29 ± 4.16 B = 1.48 ± 0.82	C = −6.90 ± 4.69 B = −2.15 ± 1.03
70 °C	C = −19.31 ± 5.77 B = −8.86 ± 14.59	C = −40.50 ± 1.11 B = −17.26 ± 10.87	C = −39.04 ± 2.02 B = −15.32 ± 11.76	C = −30.86 ± 3.58 B = −12.21 ± 12.56
80 °C	C = −15.20 ± 1.67 B = −10.54 ± 15.90	C = −26.35 ± 6.87 B = −21.38 ± 12.24	C = −34.56 ± 2.00 B = −19.01 ± 13.00	C = −34.33 ± 3.00 B = −14.79 ± 12.81
90 °C	C = −11.35 ± 3.13 B = −13.63 ± 16.24	C = −6.30 ± 8.42 B = −26.29 ± 11.56	C = −26.32 ± 2.41 B = −23.76 ± 11.82	C = −37.74 ± 4.76 B = −18.95 ± 12.76
100 °C	C = −2.48 ± 5.50 B = −10.54 ± 15.11	C = 11.59 ± 10.00 B = −22.23 ± 9.11	C = −15.94 ± 3.61 B = −20.84 ± 10.76	C = −33.77 ± 1.93 B = −18.44 ± 14.76
110 °C	C = 2.88 ± 4.28 B = −9.62 ± 9.54	C = 22.87 ± 8.82 B = −21.20 ± 4.62	C = −5.39 ± 4.73 B = −22.24 ± 7.77	C = −29.88 ± 2.09 B = −23.02 ± 14.05
